# Preliminary study of the social withdrawal (hikikomori) spectrum in French adolescents: focusing on the differences in pathology and related factors compared with Japanese adolescents

**DOI:** 10.1186/s12888-022-04116-6

**Published:** 2022-07-16

**Authors:** Yukiko Hamasaki, Nancy Pionnié-Dax, Géraldine Dorard, Nicolas Tajan, Takatoshi Hikida

**Affiliations:** 1 Faculty of Contemporary Society, Kyoto Women’s University, 35, Kitahiyoshi-cho, Imakumano, Higashiyama-ku, Kyoto, 605-8501 Japan; 2 Shigasato Hospital, 1 Chome-18-41 Shigasato, Otsu, Shiga, 520-0006 Japan; 3 Child and Adolescent Psychiatry Department, EPS ERASME, Antony, France; 4 Université Paris Cité, LPPS, F-92100 Boulogne-Billancourt, France; 5 Psychopathology and Psychoanalysis Laboratory, Graduate School of Human and Environmental Studies, Kyoto University, Kyoto, Japan; 6 Laboratory for Advanced Brain Functions, Institute for Protein Research, Osaka University, Osaka, Japan

**Keywords:** Adolescence, Comparative study, Hikikomori, Mental health, Social withdrawal

## Abstract

**Background:**

Social withdrawal (hikikomori) has become an internationally recognized phenomenon, but its pathology and related factors are not yet fully known. We previously conducted a statistical case-control study on adolescent patients with hikikomori in Japan, which revealed the non-specificity of pathology in patients with hikikomori. Further, environmental factors, such as the lack of communication between parents and Internet overuse, were found to be significant predictors of hikikomori severity. Here, we aimed to conduct a similar preliminary case-control study in France and to compare the results with those from the study conducted in Japan.

**Methods:**

Parents of middle school students who underwent psychiatric outpatient treatment for hikikomori (*n* = 10) and control group parents (*n* = 115) completed the Child Behavior Checklist to evaluate their child’s psychopathological characteristics and the Parental Assessment of Environment and Hikikomori Severity Scales, as in our previous study in Japan. We compared the descriptive statistics and intergroup differences in France with those from the previous study conducted in Japan. In the multiple regression analysis to find predictors of hikikomori severity in French and also Japanese subjects, the same dependent and independent variables were chosen for the present study (both differed from the previous study). These were used in order to make accurate intercountry comparisons.

**Results:**

The comparisons revealed no differences in the pathology of hikikomori between Japan and France. Specifically, both studies found similarly increased scores for all symptom scales, with no specific bias. However, the statistical predictors of hikikomori severity in France (lack of communication between parents and child and lack of communication with the community) differed from those in Japan (lack of communication between parents).

**Conclusion:**

Hikikomori in Japan and France could be considered essentially the same phenomenon; moreover, our findings demonstrated the universal non-specificity and unbiasedness of the hikikomori pathology. This suggests that hikikomori is not a single clinical category with a specific psychopathology; instead, it is a common phenotype with various underlying pathologies. However, different strategies may be required in each country to prevent the onset and progression of hikikomori.

## Background

Social withdrawal (hereinafter “hikikomori”) is a serious psychosocial problem in Japan since the late 1990s [1–7]. In Japan, the term “hikikomori” is used to describe both the phenomenon and a person who has stopped going to school or work and spends most of their time secluded at home.

The lifetime incidence of hikikomori in Japan is approximately 1.2% [8] with a prevalence in 15- to 39-year-olds of approximately 541,000 [9]. Hikikomori usually occurs in adolescence or early adulthood and is typically preceded by a latency period before it is clinically addressed [10]. Sociological research on this topic began in the 2000s [11–13], and in 2010, the word “hikikomori” was introduced in the Oxford English Dictionary [14]. Since then, this concept has been increasingly mentioned in the psychiatry literature [2, 4, 8, 15].

Initially, this phenomenon was considered a culture-bound syndrome unique to Japan; however, it has also been reported in other countries, including Hong Kong [16], Oman [17], Spain [18–20], France [21–24], Brazil [25], China [26], Canada [27, 28], and Italy [28]. Based on preliminary diagnostic criteria [29] and structured interviews, Teo et al. reported hikikomori cases in India, Korea, and the US [15]. Accordingly, hikikomori is currently considered a worldwide phenomenon [5, 6, 30].

Hikikomori still has no strict definition or diagnostic criteria. In 2010, the Japanese Ministry of Health, Labour, and Welfare defined hikikomori as “a state in which a person without mental illness retreats home for ≥ 6 months and does not participate in society, including attending school” [31]. Teo et al. defined hikikomori as “a period of ≥ 6 months of spending almost all the time at home and avoiding social situations and relationships, accompanied by significant distress and disability” [15]. In 2020, Kato et al. proposed diagnostic criteria for classifying people who do not meet the 6-month criterion on the severity spectrum as “pre-hikikomori”; in that study, comorbidity with other mental disorders was not an exclusion criterion [10, 32].

Hikikomori is associated with various mental disorders, including mood, anxiety, personality, developmental disorders, and the prodromal phase of schizophrenia [6, 10, 33, 34]. Additionally, there are numerous cases of hikikomori without distress (especially in the early stage), which require diagnostic attention [10, 15]. Teo et al. reported the comorbidity of withdrawal and various mental disorders, including avoidant personality disorder, social anxiety disorder, and major depression [35]. There has been increasing interest in comorbidity with social anxiety disorder [6, 9, 31, 36, 37]. Approximately 19% of patients with social anxiety disorder can be diagnosed with hikikomori [38], and approximately 18% of patients with hikikomori can be diagnosed with social anxiety disorder as well [39]. Hence, although these conditions overlap, they are not identical. The pathological features specific to hikikomori remain unclear and have not been described in the Diagnostic and Statistical Manual of Mental Disorders (DSM-5) [40] or the International Classification of Diseases (ICD-11) [41].

Since the information technology revolution, there has been increasing attention on hikikomori [42, 43]. However, whether it is a “single mental disorder” with a specific pathology or a period-specific stress-responding phenotype (such as hysteria in the late 19th and early 20th centuries) [43, 44] based on general psychiatric vulnerability should be clarified. Regarding the former point, Hayakawa et al. [45] proposed a bi-directional relationship hypothesis between oxidative stress and inflammation that could allow the identification of biomarkers for hikikomori (or its severity) and facilitate the assessment of risk factors and treatment efficacy. Contrastingly, Kato et al. suggested several general psychopathological mechanisms involved in the act of shutting in [6], which could be closely related to the latter idea.

Given the lack of epidemiological studies, factors related to the onset and severity of hikikomori in adolescents remain unclear. A Japanese study found that school dropout is the most important factor in adolescent hikikomori [46]. Specifically, there is increased school refusal and mental health problems among middle school students [47, 48]. Further, maladaptive parenting and familial dysfunction are important factors in adolescent hikikomori [49]. In Japan, hikikomori is associated with paternal absence, close contact with the mother, and lack of independence [6, 50]. The middle-school age is crucial for the early detection and intervention of hikikomori from the perspective of neurodevelopmental plasticity and malleability [51] and in terms of the aforementioned educational and family-related factors.

Accordingly, to clarify the specific pathology of hikikomori and related factors, we conducted a case-control study of adolescent patients with hikikomori (12–15 year-olds) without a DSM 4th edition text revision (DSM-IV-TR) diagnosis of Axis I disorders and mental retardation (intellectual disability) in Japan [52]. Hikikomori was diagnosed based on the definitions of the Japanese Cabinet Office Survey [9], i.e., ≥ 6 months of a person exhibiting either quasi-hikikomori (going out only to engage in hobbies) or hikikomori in a narrow sense (from rarely going out of one’s room to going out to nearby convenience stores) and the absence of schizophrenia, and physical illness. In addition, significant distress associated with social isolation was defined as an inclusion condition. We considered hikikomori severity as a spectrum and quantified it using the original hikikomori scales (Appendix 1 in [52]). We observed significantly higher symptom scale scores in the patient group than in the control group; however, none fell within the clinical range, indicating no psychiatric signs specific to hikikomori. Although psychiatric symptoms may not be considered clinically serious individually, their combination may necessitate psychiatric consultation. Furthermore, Internet overuse and lack of communication between both parents are environmental factors associated with hikikomori severity.

The present study aimed to determine whether the pathology of hikikomori and environmental factors related to the severity of hikikomori are the same in Japan and France.

## Methods

### Participants

This study was conducted in France using a similar methodology as our previous study in Japan [52]. We included psychiatric outpatients aged 12–15 years (seventh and ninth graders), who visited an adolescent outpatient clinic between December 2018 and May 2020 primarily for hikikomori treatment (*n* = 10). Additionally, we recruited a healthy control group (*n* = 115) (Table 1).

**Table 1:** Descriptive statistics and severity of hikikomori: Comparison between Japanese^*^ and French participants

	Japan^†^ Hikikomori Control	France^§^ Hikikomori Control
< Demographic variables>		
Sex (M/F)^a^	108 (66/42) 20 (10/10) 88 (56/32) NS	125 (65/60) 10 (7/3) 115 (58/57) NS^e^
Age (Mean ± SD)^b^	14.0 ± 0.9 14.1 ± 1.1 14.0 ± 0.9 NS	14.1 ± 0.8 14.3 ± 0.9 14.1 ± 0.8 NS^f^
< Hikikomori-related scales >		
Severity of absenteeism ^c^	1.22 ± 1.15	0.45 ± 0.98
Lack of going out ^d^	1.17 ± 1.10	0.88 ± 1.20

^a^ No significant difference between Japan and France. Pearson’s chi-square test. χ2 = 0.162, *p* = 0.186, ^b^ No significant difference between Japan and France. *T*-test: *t* = 0.512, *p* = 0.609, ^c^ Significant difference between Japan and France. *T*-test: *t* = 5.46, *p* < 0.001, ^d^ No significant difference between Japan and France. *T*-test: *t* = 1.94, *p* = 0.053. NS^e^: No significant difference between the hikikomori and control groups. Pearson’s chi-square test. χ2 = 1.411, *p* = 0.235. NS^f^: No significant difference between the hikikomori and control groups. Mann–Whitney *U* test: *p* = 0.363. Reliability analysis of internal consistency of each scale: ^†^ Cronbach’s α = 0.703, ^§^ Cronbach’s α = 0.316. *The Japanese sample was sourced from our previous study [52]

Among patients whose chief complaint was hikikomori, we included those who met Teo et al.’s definition of hikikomori, i.e., ≥ 6 months of a person exhibiting the following behaviors: 1) spending almost all the time at home, 2) avoiding social situations and relationships, 3) accompanied by significant distress associated with the social isolation [15]. The Japanese Cabinet Office’s definition “no schizophrenia or physical illness” was also an inclusion condition. Furthermore, we only included individuals who did not meet the criteria for Axis I mental disorders and Axis II intellectual disability (mental retardation) based on the DSM-IV-TR. These conditions consequently overlap with our previous study in Japan. All patients were screened using the Structured Clinical Interview for DSM-IV-TR Axis I Disorders (SCID-I) [53] and the Structured Clinical Interview for DSM-IV Childhood Diagnoses (Kid-SCID) [54, 55].

The healthy control group was mainly comprised of siblings of student volunteers from the Paris Descartes University, and they were recruited using the snowball sampling method (wherein respondents recommend additional eligible participants). The control and patient groups were matched for sex and age (Table 1). Similarly, we excluded controls with diagnosed physical illnesses, Axis I mental illnesses, or intellectual disability (Axis II mental retardation).

The participants received information regarding the study, and all adolescents and their parents provided consent to participate. This study was approved by the Ethics Committees of the Paris Descartes University and Kyoto Women’s University.

### Assessing the severity of hikikomori

Since hikikomori is absent in the DSM criteria, we evaluated its severity using an evaluation scale previously developed by our group (Appendix 1 in [52]), based on the definition of hikikomori and the spectrum concept proposed by the Japanese Cabinet Office [9, 36, 56, 57].

This evaluation scale comprises two items: (a) absenteeism from school and (b) going out (“the child went out either alone or with friends [unaccompanied by family members] to shop, engage in sports, and/or socialized with friends”). The items were rated by the parents on a 5-point scale from 0 (“not at all”) to 4 (“always”) for events occurring within the last 6 months. For item “b”, numerical values were scored in reverse order. Our previous study used the total score of items “a” and “b” to assess the severity of hikikomori. Reliability analysis between both items revealed a Cronbach’s alpha of 0.703, which confirmed internal consistency. However, in the present study, Cronbach’s alpha was 0.316 (Table 1). This suggests that in France, unlike in Japan, hikikomori and absenteeism do not necessarily overlap. Specifically, various factors, including the family’s economic situation, contribute to absenteeism in France [58], which differs from the situation in Japan. Therefore, only item (b) was used in the present study.

### Measuring environmental factors

To identify environmental factors related to hikikomori occurrence and severity among adolescents, the rating scale was the same as in our previous study [52] to measure parental mental health, parental physical conditions, parent-child communication, between-parent communication, parent-child conflicts, between-parent conflicts, financial status, communication with the community, and Internet overuse. Here, parents responded on a 5-point scale (Appendix 1 in [52]) considering the circumstances over the past 6 months.

### Measuring psycho-behavioral characteristics

Like our previous study, the parents completed the Child Behavior Checklist (CBCL4–18) [59, 60] to assess their children’s psycho-behavioral characteristics. The CBCL was developed by Achenbach et al. for the comprehensive assessment of children’s emotional and behavioral problems [61] and is widely used in pediatric psychiatry [62–65].

Based on the raw scores from 118 problem-behavior questions in the CBCL4–18, we calculated the scores of eight syndrome subscales (i.e., withdrawn, somatic complaints, anxious/depressed, social problems, thought problems, attention problems, delinquent behavior, and aggressive behavior), which were converted into standardized t scores based on country-specific standard values.

### Statistical analysis

Descriptive statistics and Mann–Whitney U tests were used for between-group comparisons of the t-scores of the hikikomori severity scale, nine environmental scales, and eight CBCL syndrome subscale t-scores. Effect sizes of *r* > 0.3 and > 0.5 were considered moderate and large, respectively [66].

Further, we assessed the correlations of hikikomori severity with the demographic variables (sex and age) and the nine environmental factors using Spearman’s rank correlation coefficient (*rs*). Additionally, multiple regression analysis was conducted with these as independent variables and hikikomori severity as the dependent variable to identify the significant factors contributing to hikikomori severity. We excluded variables that were weakly correlated (*rs* < 0.15) with hikikomori severity. Further, we conducted multiple regression analysis with the CBCL subscale t-score as an independent variable to clarify the pathology related to hikikomori severity. Multicollinearity verification was performed using variance inflation factor (VIF) statistics. Variables with VIF > 5 were considered multicollinear. Statistical analyses were performed using SPSS 22 software for Windows. Statistical significance was set at *p* < 0.05.

### Comparative analysis with previous Japanese research data

We compared the descriptive statistics with those of the previous study conducted in Japan (Tables 1 and 2 in [52]). The Japanese sample comprised a hikikomori patient group (*n* = 20) and a sex- and age-matched healthy control group (*n* = 88). There were no significant between-study differences in sex and age (Table 1).

**Table 2:** Between-group comparisons of the severity of hikikomori, environmental factors, and CBCL t-scores in France^†^

	Hikikomori Group	Control Group			
	Mean ± SD (SE)	Mean ± SD (SE)	*Z-*value	*p*	*r*
**<Severity of hikikomori*>***	2.50 ± .43 (0.45)	0.73 ± 1.07 (0.10)	3.836	0.000***	0.34
**<Environmental factors>**					
Parent’s psychiatric disorder	2.20 ± 1.98(0.62)	0.39 ± 1.07(0.10)	3.789	0.000***	0.34
Parent’s physical disorder	1.60 ± 1.64(0.52)	0.57 ± 1.00(0.09)	2.307	0.021*	0.21
Communication between parents and child	1.60 ± 1.34(0.42)	3.11 ± 0.95(0.08)	−3.484	0.000***	−0.31
Communication between parents	1.50 ± 1.64(0.52)	2.96 ± 1.13(0.10)	−2.768	0.006**	−0.25
Conflict between parents and child	2.40 ± 0.69(0.22)	1.57 ± 1.04(0.09)	2.399	0.016*	0.22
Conflict between parents	2.40 ± 1.77(0.56)	1.29 ± 1.03(0.09)	1.923	0.055	0.17
Economic status	2.00 ± 0.94(0.29)	2.59 ± 1.02(0.09)	−1.735	0.083	−0.16
Communication with the community	2.20 ± 1.81(0.57)	3.07 ± 1.12(0.10)	−1.558	0.119	−0.14
Overuse of the Internet	3.70 ± 0.67(0.21)	2.92 ± 1.31(0.12)	1.959	0.050	0.18
**< CBCL syndrome subscales t-scores >** ^§^					
Withdrawn	72.10 ± 9.75(3.08)	54.74 ± 6.70(0.62)	4.757	0.000***	0.43
Somatic complaints	66.60 ± 14.19 (4.49)	54.70 ± 6.08(0.56)	2.682	0.007**	0.24
Anxious/Depressed	72.50 ± 12.51(3.95)	54.83 ± 5.99(0.55)	4.265	0.000***	0.38
Social problems	61.60 ± 8.23(2.60)	53.41 ± 5.73(0.53)	3.780	0.000***	0.34
Thought problems	68.50 ± 9.45(2.99)	53.10 ± 5.94(0.55)	4.864	0.000***	0.44
Attention problems	65.90 ± 12.48(3.94)	54.97 ± 6.58(0.61)	3.342	0.001**	0.30
Delinquent behavior	66.10 ± 7.72(2.44)	54.17 ± 7.61(0.71)	4.264	0.000***	0.38
Aggressive behavior	63.80 ± 9.04(2.85)	54.37 ± 6.10(0.56)	3.699	0.000***	0.33

^†^*Mann–Whitney U test* comparisons; *p < 0.05, **p < 0.01, ***p < 0.001; *r* = Effect size; § = 70 < clinical range of the syndrome subscales. CBCL, Child Behavior Checklist

For the multiple regression analysis, we used a dependent variable different from the previous study (“going out,” as described in the section “Assessing the severity of hikikomori”). Further, we used independent variables selected in the present study for between-country comparisons.

## Results

### Descriptive statistics and between-group comparisons of hikikomori severity, environmental factors, and CBCL scores

Table 2 presents the descriptive statistics. Hikikomori severity was significantly higher in the patient group than in the control group (*p* < 0.001). With regard to environmental factors, in the hikikomori patient group, “parental psychiatric disorder” (*p* < 0.001), “parental physical disorder” (*p* < 0.05), and “conflict between parents and child” (*p* < 0.05) were significantly higher than in the control group, while “communication between parents and child” (*p* < 0.001) and “communication between parents” (*p* < 0.01) were significantly lower. However, the effect sizes of “parental physical disorder”, “communication between parents”, and “conflict between parents and child” were small (*r* < 0.3).

The patient group had significantly higher scores for all CBCL syndrome subscales than the control group (*p* < 0.01). Although the scores for “withdrawn” and “anxious/depressed” were slightly in the clinical range, no item had a large effect size (range, 0.24–0.44).

### Associations of hikikomori severity with demographic variables and environmental factors

“Parental psychiatric disorder” (*rs* = 0.243, *p* < 0.01), “parental physical disorder” (*rs* = 0.214, *p* < 0.05), “communication between parents and child” (*rs* = − 0.269, *p* < 0.01), and “communication with the community” (*rs* = − 0.235, *p* < 0.01) were significantly correlated with hikikomori severity. In the multiple regression analysis, we excluded “sex” (*rs* = 0.081), “age” (*rs* = 0.019), “conflict between parents and child” (*rs* = − 0.043), “conflict between parents” (*rs* = − 0.093), “economic status” (*rs* < 0.001), and “Internet overuse” (*rs* = 0.136). Table 3 presents the results of the multiple regression analysis. There was no multicollinearity in any of the variables. In the French sample, “communication with the community” and “communication between parents and child” showed the highest contribution to hikikomori severity. Contrastingly, these variables did not contribute to hikikomori severity in the Japanese sample, and “communication between parents” had the highest contribution.

**Table 3:** Multiple linear regression analyses with environmental factors for predicting hikikomori severity

Independent Variables	Beta	Japan^†^ *p*	VIF	Beta	France^§^ *p*	VIF
<Environmental factors>						
Parent’s psychiatric disorder	0.134	0.179	1.159	0.087	0.344	1.232
Parent’s physical disorder	−0.020	0.837	1.083	0.141	0.116	1.168
Communication between parents and child	−0.064	0.519	1.160	−0.188	0.046*	1.281
Communication between parents	−0.287	0.007**	1.275	−0.108	0.255	1.304
Communication with the community	0.019	0.852	1.224	−0.193	0.026*	1.070

*p < 0.05, **p < 0.01; †Multiple regression model statistics: R2 = 0.131. ANOVA *p* = 0.012. §Multiple regression model statistics: R2 = 0.191. ANOVA *p* < 0.001. VIF, variance inflation factor

### Associations between hikikomori severity and CBCL subscale scores

Table 4 shows the results of the multiple regression analysis using both study samples with “hikikomori severity” as the dependent variable and the eight CBCL syndrome subscale scores as independent variables. The analysis results for both samples were similar. No multicollinearity was observed in any of the variables. Among the eight independent variables, only “withdrawn” was significantly associated with hikikomori severity.

**Table 4:** Multiple linear regression analyses of CBCL subscales for predicting hikikomori severity

Independent Variables	Beta	Japan^†^ *p*	VIF	Beta	France^§^ *p*	VIF
Withdrawn	0.332	0.014*	2.508	0.313	0.006**	1.881
Somatic complaints	0.147	0.150	1.467	0.011	0.919	1.810
Anxious/Depressed	0.143	0.204	1.791	0.133	0.365	3.266
Social problems	0.100	0.430	2.293	0.134	0.243	1.995
Thought problems	0.015	0.900	1.958	0.138	0.293	2.595
Attention problems	0.078	0.559	2.538	−0.132	0.296	2.401
Delinquent behavior	−0.095	0.435	2.098	−0.054	0.596	1.555
Aggressive behavior	−0.127	0.351	2.653	−0.091	0.422	1.912

*p < 0.05, **p < 0.01; †Multiple regression model statistics: R2 = 0.310. ANOVA *p* < 0.001. §Multiple regression model statistics: R2 = 0.235. ANOVA *p* < 0.001. CBCL, Child Behavior Checklist

## Discussion

In the between-group comparisons of the Japanese sample in our previous survey, the patient group showed significantly high “parental psychiatric disorders”, “overuse of Internet”, and low “communication between parents”; the patient group showed significantly higher scores for all CBCL syndrome subscales than the control group, which were all in the subclinical range. Herein, we discuss those results in conjunction with the present results.

### Between-country comparison of the hikikomori pathology

There was no between-study difference in the pathology of hikikomori. Both studies showed similarly increased scores for all symptom scales; moreover, there was no specific bias in the appearance of symptoms. Contrarily, previous studies [6, 10, 33–35] have reported complications with various psychiatric disorders, including anxiety and mood disorders, and the prodromal phase of schizophrenia. We excluded patients with Axis I disorders according to the DSM-IV-TR, which explains the lack of mental disorders specific to hikikomori patients. Notably, the presenting symptoms were “unbiased” in both our studies. This suggests that hikikomori is not a single clinical category with a specific psychopathology; rather, it is a common phenotype with various underlying pathologies. Consistent with our findings, Kato et al. suggested the coexistence of several general psychopathological mechanisms underlying shut-in behavior [6].

In both our studies, patients with hikikomori had a significantly higher prevalence of parental psychiatric disorders, which is consistent with findings from a previous report [67] of a higher prevalence of psychiatric disorders in patients’ families. This strong genetic history suggests some psychiatric vulnerability underlying hikikomori; however, the aforementioned non-specificity of the pathology suggests that this vulnerability is unlikely to be due to a single cause specific to hikikomori in either Japan or France. Instead, all the general vulnerabilities could cause hikikomori. As previously reported [43, 68, 69], low stress tolerance and poor stress coping mechanisms in patients with hikikomori may be associated with the sum of such vulnerabilities.

Hayakawa et al. [45] proposed biomarkers for hikikomori, including oxidative stress and inflammation; however, they are more likely to be signs of physical changes that progress with hikikomori worsening rather than hikikomori-specific markers of psychiatric vulnerability. Prospective longitudinal studies are warranted to prove this hypothesis.

In this study, we quantified the severity of hikikomori in Japan and France by considering it a spectrum. We observed no specific pathology related to hikikomori severity; “withdrawn” was the only characteristic associated with hikikomori severity. Psychopathological factors may not contribute to the progression of hikikomori from the subclinical form to the clinical status; instead, there may be external factors, including the family and sociocultural background. Accordingly, the observed increased scores of the syndrome subscales may be secondary to hikikomori progression rather than a cause of its onset. Longitudinal studies are warranted to clarify the causal relationships.

### Between-country differences in factors associated with hikikomori severity

Previous studies have suggested a male predominance in withdrawal symptoms [21, 46]; however, we observed no significant correlation of sex with hikikomori severity in both countries [52].

There were between-country differences in environmental factors related to hikikomori severity. In France, “communication with the community” and “communication between parents and child” showed the highest contribution to hikikomori severity. Contrastingly, in Japan, “communication between parents” showed the highest contribution. Previous Japanese studies have reported an association of hikikomori with paternal absence, close contact with the mother, and lack of independence [6, 50, 68]. Dysfunctional family relationships have been identified in other countries, including Hong Kong, Spain, Italy, and France [22, 49, 67, 70]. Although family factors contribute to hikikomori severity worldwide, our findings suggest that these factors may differ between Europe and Japan. Specifically, parental problems (“lack of communication between parents”) and parent-child problems (“lack of communication between parents and children”) appear to be important predictors in Japan and France, respectively. Notably, both studies found that the quantity of communication, and not conflict, (which is a qualitative aspect) was an important factor. Alienation from the community was identified as a predictor in France, which was not previously reported. In France, the psychological isolation of adolescents from their families and communities may be an important factor for hikikomori. The statistical difference between the predictors of hikikomori severity in France (lack of communication between parents and child, and lack of communication with the community) and those in Japan (lack of communication between parents) might lead to diverse considerations on the roles of cultural differences and social-environment discrepancy in hikikomori. First, the combination of “lack of communication between parents and child” and “lack of communication with the community” can be considered as a “double hikikomori” where the social withdrawal of a person is associated with social isolation of his or her family [71, 72]. Second, it would mean that double hikikomori situations predict hikikomori severity in France, but not in Japan. This result might be interpreted in the light of the deep changes affecting Japanese society over the last decades [73]. To simplify, regarding hikikomori situations, while a lack of communication with the community is considered a problem in France, it is not the case in Japan. Put differently, there is still hope in France that the community can do something for hikikomori people while the attitude toward the community in Japan might be tinted with hopelessness, lack of interest, indifference, or resignation; hence the absence of “lack of communication with the community” as a predictor of hikikomori severity in Japan. Third, the fact that “lack of communication between parents” is a predictor of hikikomori severity only in Japan may be explained by differences in parental roles between the two countries. For instance, the time spent on housework and childcare by Japanese men in the general population is “at the lowest level on a global basis” [74]. Compared to other countries, Japanese fathers’ participation in the home is lower, and cooperation between parents in child rearing is also relatively low. An absent father, a subsequent mother-child closeness and over-interference, and the inhibition of children’s independence have been repeatedly mentioned in previous studies as factors in the occurrence of hikikomori in Japan [6, 50, 56]. In Japan, where generally little cooperation exists between parents, and particularly in those families where communication between parents is self-rated as relatively poorer, the above factors may surpass the threshold for triggering hikikomori. Further studies are required to assess the contribution of cultural factors in shaping parental roles, and particularly their impact on the development of hikikomori.

Our previous Japanese study [52], but not the present study, indicated an association of excessive Internet use with hikikomori severity. Stip et al. reported overuse of Internet games, social media (Instagram, Facebook, etc.), and YouTube in Asian countries, including Japan, South Korea, China, and Hong Kong, but not in other countries [28]. It remains unclear whether Internet use increases hikikomori severity or hikikomori fosters an affinity for Internet use [42]. Further research should clarify the causal relationship between the two and the influence of cultural factors.

As aforementioned, the pathology of hikikomori is non-specific and diverse. It is important to note here that there are cultural differences in factors that accelerate or mitigate hikikomori progression. Therefore, different strategies may be necessary for preventing hikikomori in different cultures. Since these factors are important for psychiatric, welfare, and educational interventions for adolescent hikikomori, further studies are warranted to determine the causal relationships of these factors with the onset and severity of hikikomori.

### Limitations of the study

This study has several limitations. First, the sample size was small; however, we decided to publish these data because we believe that the findings are valuable as a preliminary study that can guide future international studies on hikikomori. During the development of the study methodology, few studies on hikikomori had been conducted. However, we developed an ad-hoc questionnaire based on the definition of the Japanese Cabinet Office, which is a major limitation of our study. Subsequent studies have refined the definition of the hikikomori spectrum [10], and several questionnaires have been proposed (e.g., HQ-25) [75, 76]. Future studies should use standardized and validated questionnaires. Only the parents completed the questionnaire. When we started our investigation, the HQ-25 had not yet been validated. It would be interesting to collect responses from adolescents themselves regarding their social life using instruments such as the HQ-25. Further, the present study was cross-sectional; longitudinal studies are required to clarify the causal relationship between the related factors and hikikomori. This could aid the development of preventive and interventional strategies for various degrees of the hikikomori spectrum.

## Conclusion

Our findings suggest that hikikomori is not a single clinical category with a specific psychopathology; instead, it is a common phenotype with various underlying pathologies. However, the question remains as to why this phenotype is increasing worldwide. At this point, it may be reasonable to assume that the epidemic of hikikomori has emerged from various pathological bases in the backdrop of socio-familial systems that are rapidly changing due to the influence of the information technology revolution and other factors, as indicated by previous studies [5, 6, 31, 44]. Further, given the COVID-19 pandemic, there is an expected major paradigm shift in human relationships and communications as more areas of work and education move online. Consequently, it is necessary to pay attention to this hikikomori outbreak from a social psychiatric perspective in the future. It is also important to note here that there are cultural differences in factors that accelerate or mitigate hikikomori progression. Different strategies would be necessary for preventing hikikomori in different cultures. These factors are important for medical as well as welfare and educational interventions for adolescent hikikomori. Further studies are warranted to clarify the elaborate mechanisms of the onset/severity of the hikikomori phenotype, while elucidating the causal relationships among these factors, and provide clinicians with useful knowledge for early intervention across multiple fields.

## Data Availability

The data that support the findings of this study are available from the corresponding author upon reasonable request. The assessment scales and the results of the descriptive statistics of the previous study conducted in Japan are available at https://link.springer.com/article/10.1007/s10578-020-01064-8 [52].

## Abbreviations

CBCL4–18: Child Behavior Checklist/4–18
COVID-19: Coronavirus disease
DSM-IV-TR: Diagnostic and Statistical Manual of Mental Disorders, 4th edition, text revision
DSM-5: Diagnostic and Statistical Manual of Mental Disorders, 5th edition
HQ-25: 25-item Hikokimori Questionnaire
ICD-11: International Classification of Diseases, 11th revision.
VIF: Variance inflation factor
